# Activity and Safety of Immune Checkpoint Inhibitors in Neuroendocrine Neoplasms: A Systematic Review and Meta-Analysis

**DOI:** 10.3390/ph14050476

**Published:** 2021-05-17

**Authors:** Alberto Bongiovanni, Brigida Anna Maiorano, Irene Azzali, Chiara Liverani, Martine Bocchini, Valentina Fausti, Giandomenico Di Menna, Ilaria Grassi, Maddalena Sansovini, Nada Riva, Toni Ibrahim

**Affiliations:** 1 Osteoncology and Rare Tumors Center (CDO-TR), IRCCS Istituto Romagnolo per lo Studio dei Tumori (IRST) “Dino Amadori”, 47014 Meldola, Italy; brigidamaiorano@gmail.com (B.A.M.); chiara.liverani@irst.emr.it (C.L.); valentina.fausti@irst.emr.it (V.F.); giandomenico.dimenna@irst.emr.it (G.D.M.); nada.riva@irst.emr.it (N.R.); toni.ibrahim@irst.emr.it (T.I.); 2 Oncology Unit, Foundation Casa Sollievo della Sofferenza IRCCS, 71013 San Giovanni Rotondo, Italy; 3 Unit of Biostatistics and Clinical Trials, IRCCS Istituto Romagnolo per lo Studio dei Tumori (IRST) “Dino Amadori”, 47014 Meldola, Italy; irene.azzali@irst.emr.it; 4 Immunotherapy, Cell Therapy and Biobank (ITCB), IRCCS Istituto Romagnolo per lo Studio dei Tumori (IRST) “Dino Amadori”, 47014 Meldola, Italy; martine.bocchini@irst.emr.it; 5 Nuclear Medicine Unit, IRCCS Istituto Romagnolo per lo Studio dei Tumori (IRST) “Dino Amadori”, 47014 Meldola, Italy; ilaria.grassi@irst.emr.it (I.G.); maddalena.sansovini@irst.emr.it (M.S.)

**Keywords:** immune checkpoint inhibitors, neuroendocrine tumors, PD1, PD-L1

## Abstract

Immune-checkpoint inhibitors (ICIs) have widened the therapeutic scenario of different cancer types. Phase I/II trials have been designed to evaluate the role of ICIs both as single agents and in combination in neuroendocrine neoplasms (NENs), but as yet no randomized controlled phase III trials have been carried out. A systematic review and meta-analysis of studies published could help to reduce the biases of single-phase II trials. Efficacy data were obtained on 636 patients. Pooled percentages of the overall response rate (ORR) and disease control rate (DCR) were 10% (95% CI: 6–15%, I^2^ = 67%, *p* < 0.1) and 42% (95% CI: 28–56%, I^2^ = 93%, *p* < 0.1), respectively. Median progression-free survival (mPFS) was 4.1 months (95% CI 2.6–5.4; I^2^ = 96%, *p* < 0.1) and median overall survival (mOS) was 11 months (95% CI 4.8–21.1; I^2^ = 98%, *p* < 0.1). Among the ICIs used as single agents, the anti-PD1 toripalimab achieved the highest ORR. Combination regimens were superior to monotherapy, e.g., the ICI combination nivolumab + ipilimumab, and the ICI + anti-angiogenetic combination atezolizumab + bevacizumab, both of which warrant further investigation. Promising efficacy and a good safety profile of ICIs represent a valid opportunity for expanding the therapeutic landscape of NENs. Predictive biomarkers are needed to identify the most suitable candidates for these regimens.

## 1. Introduction

Neuroendocrine neoplasms (NENs) are a heterogeneous group of tumors originating from neuroendocrine cells [1,2]. NENs can present at different sites, the most frequent being the gastroenteropancreatic compartment (up to 70% of all tumors) and respiratory tract (around 20%), while other regions such as the genitourinary and gynecological tracts are less frequently involved. The incidence of NENs has increased in recent years and is now estimated to be around 2.5–5 cases per 100,000 inhabitants/year in Europe and the U.S. [2,3,4]. The therapeutic approach to NENs is based on primary tumor location, grading, and staging [5]. In unresectable lesions, the mainstays of treatment are “cold” somatostatin analogs (SSAs), peptide receptor radionuclide therapy (PRRT), tyrosine kinase inhibitors (TKIs), and the mammalian target of rapamycin (mTOR) inhibitor, everolimus [5,6,7,8,9,10,11,12,13,14,15,16]. Chemotherapy also plays a role, especially for more aggressive lesions or high-grade and poorly-differentiated NENs [17,18,19,20].

In many cancer types, immunotherapy with immune checkpoint inhibitors (ICIs) has dramatically changed the natural history of the disease [21]. ICIs are monoclonal antibodies that block the negative signal for the activation of effector T-cells, thereby de-inhibiting the anti-tumor immune response [22]. To date, the main pathways targeted by ICIs are programmed cell-death protein 1 (PD-1)/programmed death-ligand 1 (PD-L1) and cytotoxic T-lymphocyte antigen-4 (CTLA-4). Several ICIs have already been approved for clinical use or are under investigation in various cancer types. For example, nivolumab, pembrolizumab, toripalimab, and spartalizumab act as anti-PD1 agents; avelumab, atezolizumab, and durvalumab are directed against PD-L1; and ipilimumab and tremelimumab bind to CTLA-4. These drugs can be used both as single agents and as combinations of anti-PD1/PD-L1 plus anti-CTLA4 [23,24,25]. However, as the immunological features of NENs are not fully understood, there is still limited evidence of the efficacy and safety of ICIs in these malignancies, mainly represented by phase I and II trials [26,27,28,29,30,31,32,33,34,35,36,37,38,39].

According to the latest WHO classification, gastroenteropancreatic G3 NENs are defined by a Ki67 proliferation index of >20%. In addition to proliferative activity, tumor differentiation appears to play a major role, further dividing the G3 NEN G3 group into G3 neuroendocrine tumors (NETs) and NECs, characterized by different prognosis and response to available therapies [40,41].

In the present systematic review and meta-analysis, we summarize what is currently known about the efficacy and safety of ICIs in NENs, and the possible implications for clinical practice.

## 2. Materials and Methods

### 2.1. Data Extraction

**Figure 1:**
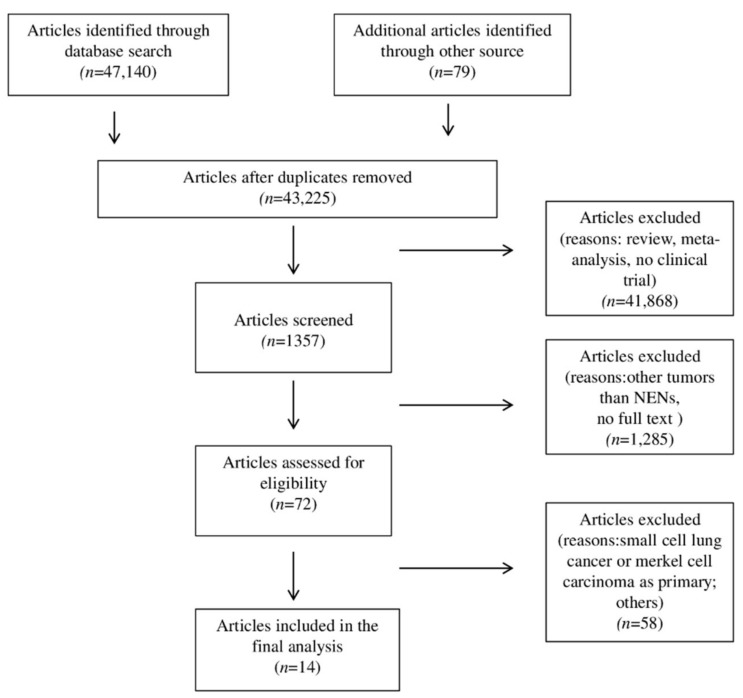
Flow diagram of search methods.

This systematic review and meta-analysis were conducted in accordance with the guidelines of the Preferred Reporting Items for Systematic Reviews and Meta-Analyses (PRISMA) [42,43]. A search of major databases, e.g., MEDLINE/PubMed, Cochrane, and Embase, was performed to identify studies on ICIs for the treatment of NENs published up to October 2020. The following keywords were used: “immune checkpoint inhibitors” or “ICIs” or “nivolumab” or “pembrolizumab” or “durvalumab” or “avelumab” or “atezolizumab” or “spartalizumab” or “toripalimab” or “tremelimumab” or “ipilimumab” or “anti PD1” or “anti PD-L1” or “anti-CTLA4” and “neuroendocrine tumors” or “neuroendocrine neoplasms” or “neuroendocrine carcinomas”. An additional search for Meeting Abstracts published by the American Society of Clinical Oncology (ASCO), ASCO Gastrointestinal Symposium (ASCO-GI), European Society for Medical Oncology (ESMO), and European Neuro-Endocrine Tumors Society (ENETS) annual meetings was also performed. Finally, we manually checked the citations of the included publications. Original studies published in English were considered. Reviews, letters, and personal opinions were excluded. A flowchart of the selection process is shown in Figure 1.

Two authors independently conducted a preliminary screening of titles and abstracts, followed by a second-round screening when they read the full texts of the potentially relevant articles. In the event of disagreement, a third reviewer was consulted to facilitate the final decision. Data on the population treated, treatment efficacy, and toxicity parameters were extracted and pooled from the selected studies. The following data were also collected from each study: year of publication, name of first author, country of the study; study design (phase, randomization, independent review); baseline characteristics of the included patients (median age, histologic classification, grading, staging, primary tumor location); intervention including treatment regimens, dosage and number of administered cycles; complete response (CR), partial response (PR), stable disease (SD) and progressive disease (PD) absolute frequency or, when available, outcomes expressed as overall response rate (ORR) and disease-control rate (DCR); progression-free survival (PFS) and overall survival (OS); toxicities (grades and types of trAEs and irAEs). When response and toxicities were reported at the subgroup level, the values were combined to have endpoints at the single-cohort level. PFS and OS were maintained at the subgroup level because their nature (medians) does not allow for an overall merging value.

### 2.2. Quality Assessment

The risk of bias was assessed by the Risk Of Bias in Non-randomized Studies of Interventions (ROBINS-I) tool for non-randomized clinical trials [44]. In this tool, risk of bias is assessed within specified domains, including (1) bias due to confounding, (2) bias in the selection of participants for the study, (3) bias in the classification of interventions, (4) bias due to deviations from intended interventions (5) bias due to missing data, (6) bias in the measurement of outcomes, (7) bias in the selection of the reported results, and (8) overall bias. Given that assessments are inherently subjective and that there are no strict or objective criteria to judge bias within the ROBINS-I tool, disagreements were resolved by the intervention of a third investigator.

### 2.3. Statistical Analysis

The main endpoints of ORR, DCR, any trAE, any irAE, trAEs of grade ≥3, and irAEs of grade ≥3 were pooled together using the Meta package in R software (version 3.6.1). Relative risks and 95%CIs describing the ORR of PD-L1 biomarkers were synthesized calculated using the Meta package in R. In addition, the median PFS and OS values, accompanied by their 95%CIs, were extracted and pooled estimates obtained according to the method used by McGrath et al., [45]. This meta-analysis was performed in R software using the metamedian package. Heterogeneity between study outcomes was evaluated using the I^2^ index and Cochrane’s Q-test (reported with *p*-value). Values of I^2^ > 50% and *p* < 0.1 indicated a substantial heterogeneity between studies. The pooled estimates with their 95% CI were determined using the fixed-effects model (*p* > 0.1) or the random-effects model (*p* < 0.1). While the Meta R package automatically performs the I^2^ and Q-test calculus, ad hoc code was written to evaluate the I^2^ and Q-test on PFS and OS. Subgroup analyses of primary efficacy endpoints were included to investigate the possible sources of heterogeneity and to identify differences in subsets of patients. A funnel plot of the main endpoint ORR was generated to assess potential publication bias, and its asymmetry was evaluated via linear regression test using the Meta package in R software (3.6.1).

## 3. Results

### 3.1. Characteristics of Studies

A total of 14 studies fulfilled selection criteria and were included in the systematic review and meta-analysis (Figure 1): 7 were peer-reviewed full-text publications from scientific journals [26,27,29,30,31,36,38] and 7 were conference abstracts or posters [28,32,33,34,35,37,39]. The studies in question were all non-randomized, prospective studies, 3 of which phase Ib [26,30,33]. The remaining 11 studies were phase II trials [27,28,29,31,32,35,36,37,38,39]. The majority of studies (11/14) were multicenter. An independent review was declared for 4 studies [27,28,32,39]. The main characteristics of the included studies are reported in Table 1. We differentiated between neuroendocrine tumors (NETs) and neuroendocrine carcinoma (NECs) when this was specified by the authors, and indicated cases not specifically identified as NETs or NECs as neuroendocrine neoplasms (NENs).

### 3.2. Patient Characteristics at Baseline

Six hundred and thirty-six patients were treated with ICIs either as monotherapy or in combination. The median age at enrolment ranged across studies from 41 to 67 years. Eastern Cooperative Oncology Group performance status (ECOG PS) at screening was 0–1 in 10/14 studies and 0–2 in the remaining 4. The most frequent site of origin of NENs was the pancreas (219 patients, 34.2%) followed by the gastrointestinal tract (201 patients, 31.4%) and lung (100 patients, 15.6%). The remaining 72 (11.3%) patients had NENs originating from other sites or of unspecified/unknown origin. The majority of patients (418, 65.3%) had grade (G) 1 or G2 NENs. Eighty-six (13.4%) patients had G3 neuroendocrine tumors (NETs) and 114 patients (17.8%) had neuroendocrine carcinomas (NECs); in 13 (2%) cases, the distinction between NET G3 and NEC was not specified. Grading was unknown in 9 (1.5%) cases.

The patients included in the studies were all pre-treated, but data on previously administered therapies were available in only 3 studies for a total of 193 patients [26,38,39]. Somatostatin analogs (SSAs) were the most widely used drugs (90 patients), followed by chemotherapy (110 patients), everolimus (76 patients), and TKIs (26 patients). Functional status was not mentioned in just under half of the studies (6/14). In the 8 articles in which this information was given, all the patients had non-functioning tumors, except the study by Strosberg et al. that included 8 functioning tumors [27].

Among ICIs targeting PD-1, 198 (31%) patients received pembrolizumab as a single agent, 116 (18.1%) were treated with spartalizumab and 63 (9.8%) with toripalimab. Thirty-nine (6.1%) patients received the anti-PD-L1 avelumab as a single agent.

The combination of anti-PD1 and anti-CTLA4 drugs was administered as durvalumab plus tremelimumab in 123 (19.2%) cases, or as nivolumab plus ipilimumab in 61 (9.5%) patients. Forty (6.3%) patients received a combination of anti-PD-L1 atezolizumab with the anti-vascular endothelial growth factor (VEGF) bevacizumab.

In 7 studies, 370 patients were evaluated for PD-L1 expression [26,27,30,31,33,36,39]. However, a PD-L1 positivity of ≥1% was an inclusion criterion in only one study [26]. ORR was the main primary endpoint, while secondary endpoints were DCR, OS, PFS, and safety.

### 3.3. Clinical Outcomes

**Figure 2:**
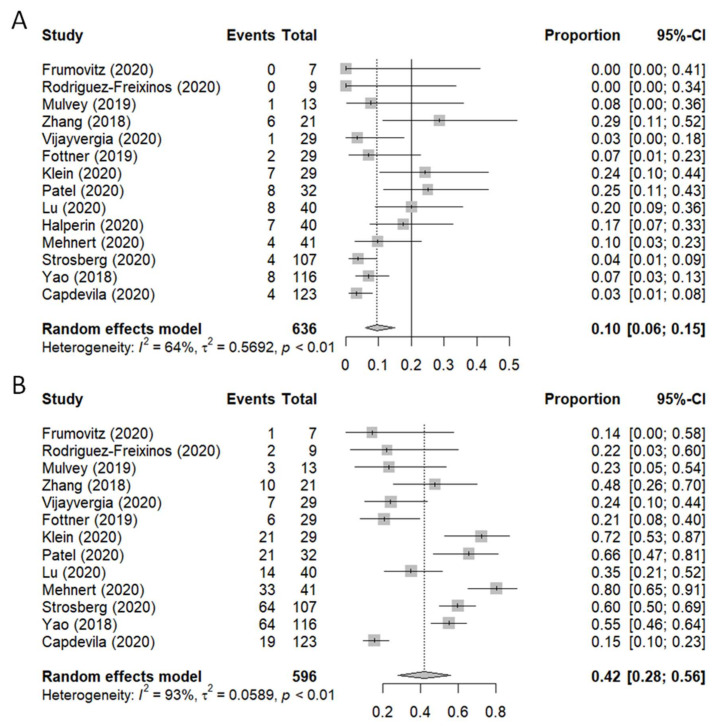
Forest plot of (**A**) overall response rate (ORR) and (**B**) disease control rate.

Figure 2 shows the forest plots of ORR (A) and DCR (B), reported as percentages, along with their 95% confidence interval (95%CI). The efficacy analysis of the ORR was carried out on 636 patients, while only 596 patients were included in the DCR analysis. In one study, information on DCR or stable disease was not available [32]. The best response obtained was a partial response (PR) in 39 patients and stable disease (SD) in 210 patients. Three complete responses (CR) were registered. The pooled proportions of ORR and DCR were 0.10 (95% CI: 0.06–0.15, I^2^ = 67%, *p* < 0.1) and 0.42 (95% CI: 0.28–0.56, I^2^ = 93%, *p* < 0.1), respectively. The linear regression test (*p* = 0.212) indicated that no publication bias existed in this meta-analysis for ORR (Figure S1).

### 3.4. PD-L1 Biomarker for ORR

**Figure 3:**
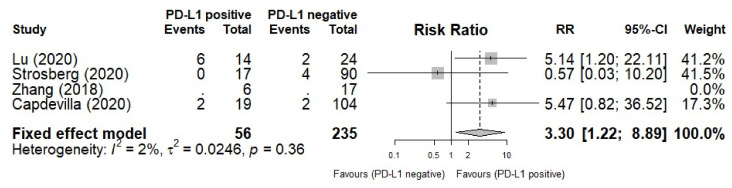
Forest plot of overall response rate (ORR) based on PD-L1 expression.

Four studies with PD-L1 expression data were included in an additional analysis to analyze the prognostic role of PD-L1 for ORR. Figure 3 shows the forest plots of ORR comparisons based on PD-L1 expression. PD-L1-positive patients had a higher ORR than those with PD-L1 negative tumors (RR = 1.22, 95% CI: 1.22–8.89, I^2^ = 2%, *p* = 0.36).

### 3.5. Subgroup Efficacy Analysis: ORR and DCR

**Figure 4:**
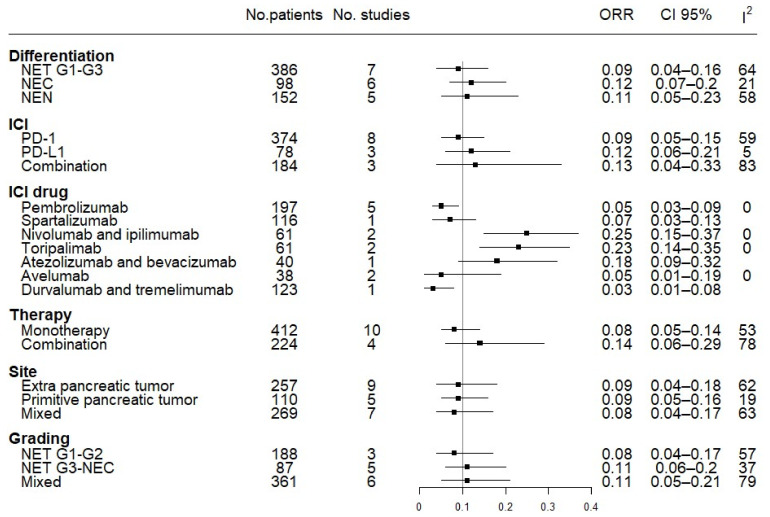
Forest plot of overall response rate (ORR) analysis by subgroups. Different tumor differentiation, ICI targets, ICI drugs, type of therapy (mono versus combo), site of the primary tumor, and grading have been grouped together.

For the subgroup analyses of the available data, we first grouped the studies according to the different mechanisms of action of the ICIs used (anti-PD1 monotherapy vs. anti-PD-L1 monotherapy vs. combination anti-PD1/PD-L1 + anti-CTLA4/anti-VEGF therapy), observing a general heterogeneity of the studies (I^2^ = 67%) and a trend of better ORR for combination regimens (0.13, 95% CI 0.06–0.29) compared to single agents (0.08, 95% CI 0.05–0.14). We then analyzed the effect of the specific drugs on the primary outcome (ORR). Among the anti-PD1 agents, toripalimab obtained the best results in terms of ORR (0.23, 95% CI 0.14–0.35), while pembrolizumab and spartalizumab produced the poorest results (0.05, 95% CI 0.03–0.09 and 0.07, 95% CI 0.03–0.13, respectively). Anti-PD1 agents also obtained the best results for ORR as a combination of nivolumab + ipilimumab (0.25, 95% CI 0.15–0.37). The drugs directed against PD-L1 showed a poorer performance, i.e., single-agent avelumab (0.05, 95% CI 0.01–0.19) and the combination of durvalumab + tremelimumab (0.03, 95% CI 0.01–0.08). One exception was the combination of the anti-PDL1 atezolizumab and the anti-VEGF bevacizumab, which obtained a higher ORR (0.18, 95% CI 0.09–0.32). There were no differences between NENs originating from the pancreas and those originating from extra-pancreatic sites (Figure 4).

**Figure 5:**
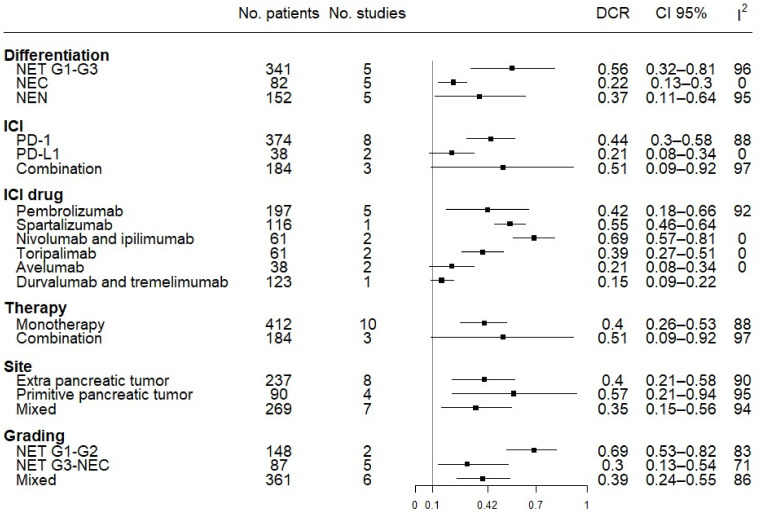
Forest plot of DCR analysis by subgroups. Different tumor differentiation, ICI targets, ICI drugs, type of therapy (mono versus combo), site of primary tumor, and grading have been grouped together.

The DCR subgroup analysis confirmed the same trend for ORR in the above subgroups. Anti-PD1 monotherapy and combination regimens showed a higher DCR (0.44, 95% CI 0.3–0.58 and 0.51, 95% CI 0.09–0.92, respectively) than single agents directed against PD-L1 (0.21, 95% CI 0.08–0.34), with better results when spartalizumab (0.55, 95% CI 0.46–0.64) and nivolumab + ipilimumab (0.69, 95% CI 0.57–0.81) were used. Of note, when grouping the studies according to differentiation grade, NETs showed better results (0.56, 95% CI 0.32–0.81) than NECs (0.22, 95% CI 0.13–0.30). With regard to tumor grade, G1/G2 NETs had a higher DCR (0.69, 95% CI 0.53–0.82) than G3 NETs/NECs (0.3, 95% CI 0.13–0.54). Furthermore, for DCR, differences among NEN sites of origin were not significant for the DCR (Figure 5).

### 3.6. PFS and OS

**Figure 6:**
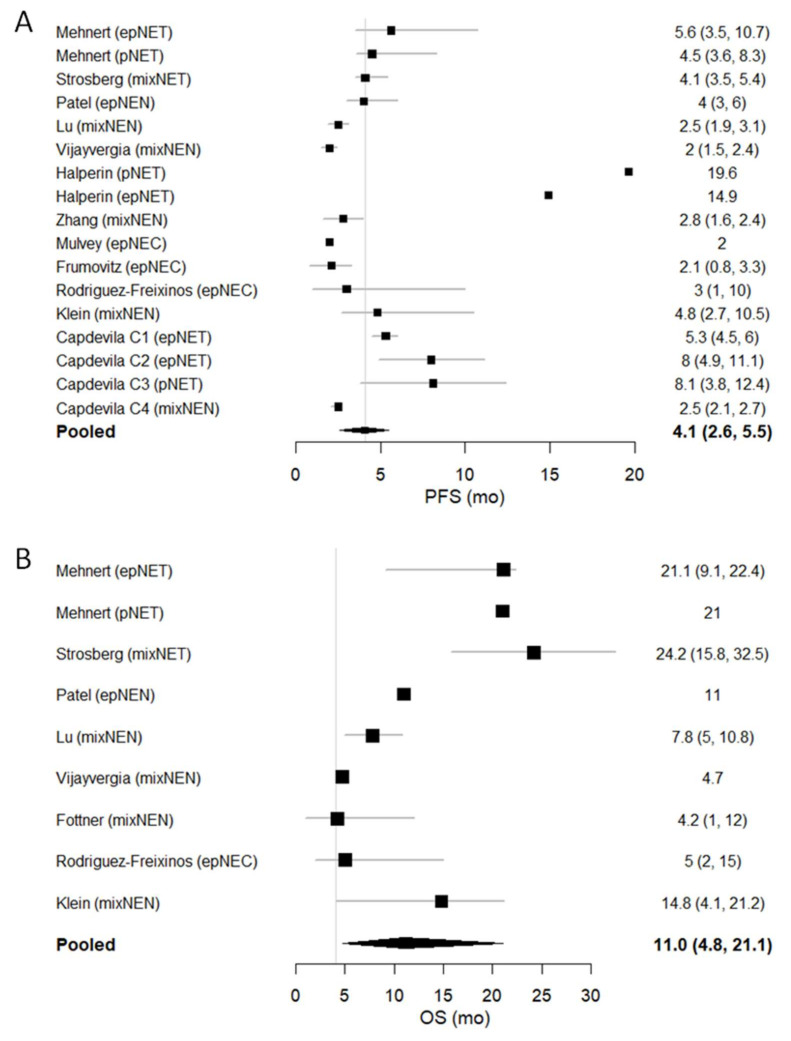
Forest plot of (**A**) median PFS and (**B**) median OS.

The forest plots of PFS and OS (reported as months), together with their 95% CI (whenever available), are shown in Figure 6. We conducted a pooled analysis of PFS for the 12 studies in which these data were reported [26,27,30,31,32,33,35,36,37,38,39]. The pooled median PFS (mPFS) was 4.1 months (95% CI 2.6–5.4; I^2^ = 96%, *p* < 0.1). Of note, the combination of atezolizumab + bevacizumab achieved the longest mPFS (19.6 months in the pancreatic NET cohort and 14.9 months in the extra-pancreatic NET cohort). Data for OS were available in 8 studies [26,27,29,30,31,34,37,38]. The pooled median OS (mOS) from the trials was 11 months (95% CI 4.8–21.1; I^2^ = 98%, *p* < 0.1).

### 3.7. Safety Analysis

**Figure 7:**
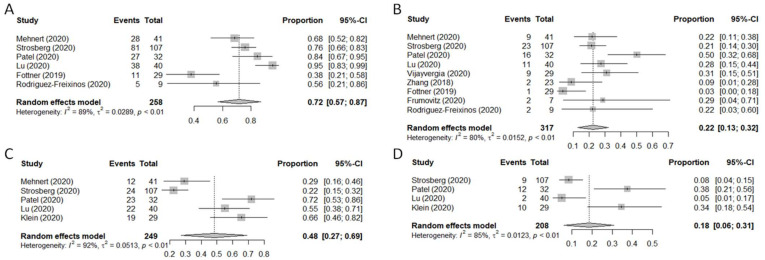
Forest plot of safety endpoints. Treatment-related adverse events (trAEs) of any grade (**A**) or ≥grade 3 (**B**). Immune-related adverse events (irAEs) of any grade (**C**) or ≥grade 3 (**D**).

Ten of the 14 studies reported safety outcomes (Table 2) [26,27,29,30,31,33,34,36,37,38]. The rate of all-grade toxicities ranged from 38% to 95%. In most cases, the distinction between tumor-related adverse events (trAEs) and immune-related adverse events (irAEs) was listed. these, the most frequent adverse events (AEs) were dermatologic toxicities (rash, dermatitis, pruritus; *n* = 135), fatigue (*n* = 132), gastrointestinal side-effects such as nausea/vomiting (*n* = 86) or diarrhea (*n* = 84), decreased appetite (*n* = 24) or, more rarely, weight loss (*n* = 7) or colitis (*n* = 9). Sixty-nine cases of increased transaminase levels were observed, and 53 patients developed hypothyroidism. G3-G4 AEs were registered in 82 patients, which led to treatment discontinuation in 32 cases. The pooled analysis carried out on treatment-related AEs (trAEs) and immune-related AEs (irAEs) revealed a 72% frequency of any-grade trAEs (95% CI: 57–87%, I^2^ = 89%, *p* < 0.1), a 22% frequency of trAEs ≥G3 (95% CI: 13–32%, I^2^ = 80%, *p* < 0.1), a 48% frequency of any-grade irAEs (95% CI: 27–69%, I^2^ = 92%, *p* < 0.1) and an 18% frequency of irAEs ≥G3 (95% CI: 6–31%, I^2^ = 85%, *p* < 0.1) (Figure 7).

### 3.8. Risk of Bias

All the included non-randomized clinical trials were considered to be at low risk of bias, except the study by Halperin et al., which was deemed to have a moderate risk of bias [32]. More details on the risk of bias assessment, with reasons supporting each study assessment, can be found in Supplementary Table S1.

## 4. Discussion

In recent years, research into immuno-oncology has led to important breakthroughs in the treatment of solid tumors [21]. Although some immunological therapies, such as interferon, have already been used successfully in patients with NETs, the role of ICIs for NENs has yet to be clarified [5]. Several phase Ib/II trials recently explored single-agent and combination therapy with ICIs in this disease setting. However, the “one-fits-all” approach, albeit useful to reach the planned study accrual due to the rarity of the disease, has not provided conclusive results [46]. Our meta-analysis confirmed the heterogeneity of results across studies as a consequence of the clinical characteristics of enrolled patients (i.e., primary site, grade, percentage of metastatic cases) and the type of samples analyzed. It also enabled us to identify future directions for research into the use of ICIs in NENs.

In our meta-analysis, anti-PD1 compounds such as toripalimab produced an ORR of 23%, while the combination of anti-PD1 nivolumab with anti-CTLA4 ipilimumab reached 25% ORR in a mixed population in 2 studies [29,33,38]. Conversely, anti-PDL1 therapies generally failed in their main objective [29,37,39], which might be attributable to the heterogeneity of PD-ligand expression, PD-L1 being prevalent in lung NETs and PD-L2 in pNETs, suggesting a different clinical resistance to PD-1/PD-L1 checkpoint inhibitors [47]. The ORR was higher in PDL1 positive patients than in PDL1-negative cases.

Neo-angiogenesis appears to play a key role in NEN development and progression. NETs are, in fact, characterized by a high vascular supply and overexpression of VEGF-A. The latter induces immunosuppressive cells, e.g., tumor-associated macrophages, regulatory T cells, and myeloid-derived suppressor cells, leading to an immunosuppressive phenotype that inhibits the maturation of dendritic cells and the activation and proliferation of T cells [48]. Thus, treatment with an anti-VEGF antibody could reverse these immunosuppressive effects and promote T cell activation and dendritic cell maturation. In fact, in the study by Halperin et al., the addition of bevacizumab to atezolizumab led to an ORR of 15–20% and a PFS of 14.9–19.6 months in extra-pancreatic and pancreatic NETs, suggesting the synergistic activity of bevacizumab in converting an immune ‘cold’ tumor into a ‘hot’ one [32].

Another important issue is the short-term PFS obtained from the use of ICIs (except for Halperin’s study), similar to that observed in the placebo arms of studies on everolimus and sunitinib [12,15]. A possible explanation for this could be that ICIs are more beneficial in the early phase of the natural history of NENs, as observed in Halperin’s study. This benefit may be lost in heavily pre-treated patients due to modifications occurring in both the tumor and the microenvironment. In addition to the need for larger studies, a potential future strategy could involve the use of anti-PD1 therapy, perhaps in combination with antiangiogenic therapy, in patients selected based on grading and site of origin.

The safety profile of ICIs is also an important aspect to be considered. Our analysis revealed a range of side effects that are consistent with those previously reported in other solid tumors.

Our study has a number of limitations. First, all of the studies selected were non-randomized phase I/II trials. Furthermore, to avoid publication bias, we were obliged to include studies characterized by high population heterogeneity because of the poor accrual potential for a rare disease such as neuroendocrine neoplasia. Thus, although the meta-analysis did not yield conclusive results on the use of ICIs in NENs, it nevertheless shed light on interesting therapeutic possibilities such as the use of ICI combinations with anti-VEGF or anti-CTLA-4 therapies. Of note, even if we had conducted a comprehensive literature search with a sensitive search algorithm and an extensive manual search of reference lists and conference proceedings, we would not have been able to find unpublished data on every specific site of origin of the disease. We are fully aware that a substantial amount of information is not available to the public and, as such, cannot completely rule out publication bias.

## 5. Conclusions

Overall, our meta-analysis of data on ICIs in NENs revealed a high heterogeneity of treatment response. Although our results are not conclusive, they suggest that ICIs are active in NENs. Some information obtained on the different activity of anti-PD1 and anti-PDL1, and on the association with other compounds (i.e., anti-CTLA4 or anti-VEGF) could be useful for the design and development of future clinical trials.

## Figures and Tables

**Table 1:** Principal characteristics of phase II studies.

Study	Phase	Design	ICI	Target	Sample Size *	ORR No. (%)	Median PFS, mos (Range)	Median OS, mos (Range)
Mehnert [26] (NCT02054806)	1b	Multicohort: epNET	Pembrolizumab	PD-1	25	3 (12)	5.6 (3.5–10.7)	21.1 (9.1–22.4)
Mehnert [26] (NCT02054806)	1b	Multicohort: pNET	Pembrolizumab	PD-1	16	1 (6)	4.5 (3.6–8.3)	21.0
Strosberg [27] (NCT02628067)	2	Single cohort: mixNET	Pembrolizumab	PD-1	107	4 (4)	4.1 (3.5–5.4)	24.2 (15.8–32.5)
Yao [28] (NCT02955069)	2	Multicohort: epNET	Spartalizumab	PD-1	62	6 (10)	-	-
Yao [28] (NCT02955069)	2	Multicohort: pNET	Spartalizumab	PD-1	33	1 (3)	-	-
Yao [28] (NCT02955069)	2	Multicohort: mixNEC	Spartalizumab	PD-1	21	1 (5)	-	-
Patel [29] (NCT02834013)	2	Single cohort: epNEN	Nivolumab + ipilimumab	PD-1, CTLA-4	32	8 (25)	4.0 (3.0–6.0)	11
Lu [30] (NCT03167853)	1b	Multicohort: mixNEC, mixNET/pNEN, epNEN, mixNEN	Toripalimab	PD-1	40	8 (20)	2.5 (1.9–3.1)	7.8 (5.0–10.8)
Vijayvergia [31] (NCT02939651)	2	Single cohort: mixNEN	Pembrolizumab	PD-1	29	1 (3)	2.0 (1.5–2.4)	4.7
Halperin [32] (NCT03074513)	2	Multicohort: pNET	Atezolizumab + bevacizumab	PD-L1, TKI	20	4 (20)	19.6	-
Halperin [32] (NCT03074513)	2	Multicohort: epNET	Atezolizumab + bevacizumab	PD-L1, anti-VEGF	20	3 (15)	14.9	-
Zhang [33] (NCT03167853)	1b	Multicohort: mixNEC, mixNET	Toripalimab	PD-1	21	6 (29)	2.8 (1.6–4.0)	-
Fottner [34] (NCT03352934)	2	Single cohort: mixNEN	Avelumab	PD-L1	29	2 (7)	-	4.2 (1.0–12.0)
Mulvey [35] (NCT03136055)	2	Single cohort: epNEC	Pembrolizumab	PD-1	13	1, 8	2.0	-
Frumovitz [6] (NCT02721732)	2	Single cohort: epNEC	Pembrolizumab	PD-1	7	0 (0)	2.1 (0.8–3.3)	-
Rodriguez-Freixinos [37] (NCT03278405)	2a	Single cohort: epNEC	Avelumab	PD-L1	9	0 (0)	3.0 (1.0–10.0)	5.0 (2.0–15.0)
Klein [38] (NCT02923934)	2	Single cohort: mixNEN	Nivolumab + ipilimumab	PD-1, CTLA-4	29	7 (24)	4.8 (2.7–10.5)	14.8 (4.1–21.2)
Capdevila [39] (NCT03095274)	2	Multicohort:epNET	Durvalumab + tremelimumab	PD-L1, CTLA-4	27	0 (0)	5.3 (4.5–6.0)	-
Capdevila [39] (NCT03095274)	2	Multicohort:epNET	Durvalumab + tremelimumab	PD-L1, CTLA-4	31	0 (0)	8 (4.9–11.1)	-
Capdevila [39] (NCT03095274)	2	Multicohort:pNET	Durvalumab + trremelimumab	PD-L1, CTLA-4	32	2 (6)	8.1 (3.8–12.4)	-
Capdevila [39] (NCT03095274)	2	Multicohort:mixNEN	Durvalumab + tremelimumab	PD-L1, CTLA-4	33	2 (6)	2.5 (2.1–2.7)	-

* The sample size refers solely to patients evaluable for response. ICI: immune checkpoint inhibitor; ORR: objective response rate; PFS: progression-free survival; OS: overall survival; mos: months; ep: extra-pancreatic; p: pancreatic; mix: pancreatic and extra-pancreatic.

**Table 2:** List of side-effects (trAEs and irAEs) grouped by grade.

Adverse Events	trAEs (No. Cases)		irAEs (No. Cases)	
	Any Grades	≥Grade 3	Any Grades	≥Grade 3
Adrenal insufficiency	0	0	0	0
Anorexia	23	1	23	1
Arthralgia/arthritis	10	1	10	1
Asthenia	12	1	12	1
Colitis/ulcerative colitis/peritonitis	6	6	6	6
Diarrhea	67	12	67	12
Dyspnea	5	2	5	2
Electrolyte alterations	17	3	17	3
Elevated alkaline phosphatase	11	6	11	6
Elevated AST/ALT	46	11	46	11
Elevated lipase/amylase, pancreatitis	18	6	18	6
Fatigue	123	7	123	7
Fever	11	0	11	0
Hematologic alterations (anemia, decrease in white blood cells/platelets)	41	3	41	3
Hepatitis	2	2	2	2
Hyperbilirubinemia	16	2	16	2
Hyperglycemia, diabetes mellitus	18	5	18	5
Hyperthyroidism	0	0	0	0
Hypoalbuminemia	7	0	7	0
Hypophysitis	1	0	1	0
Hypotension/hypertension	4	2	4	2
Hypothyroidism	27	1	27	1
Infusion-related reactions	1	0	1	0
Muscular adverse events (weakness, myalgia, increased CK)	14	1	14	1
Myocarditis	0	0	0	0
Nausea/vomiting	57	2	57	2
Pain (abdomen, head)	7	0	7	0
Pneumonitis	1	1	1	1
Proteinuria	28	0	28	0
Retinopathy, encephalopathy	0	0	0	0
Skin toxicity (rash, dermatitis, worsening psoriasis, pruritus)	89	5	89	5
Weight loss	7	0	7	0
Other gastrointestinal toxicity (dizziness, dry mouth, dysgeusia)	7	0	7	0

AST: aspartate aminotransferase; ALT: alanine aminotransferase; CK: creatine kinase.

## Data Availability

The datasets generated and/or analyzed during the current study are available from the corresponding author on reasonable request.

## Supplementary Materials

The following are available online at https://www.mdpi.com/article/10.3390/ph14050476/s1, Figure S1: Funnel plot of overall response rate (ORR) for publication bias, Table S1. Risk of bias for non-randomized studies.
