# Supplementary material for: Activity and Safety of Immune Checkpoint Inhibitors in Neuroendocrine Neoplasms: A Systematic Review and Meta-Analysis
Source: Pharmaceuticals (Basel). 2021 May 17;14(5):476. doi: 10.3390/ph14050476 (PMC8155858; doi:10.3390/ph14050476)
Supplement: Supplementary file 1 [file pharmaceuticals-14-00476-s001.zip › pharmaceuticals-1218854-supplementary.pdf]

SUPPLEMENTARY MATERIALS

Table S1. Risk of bias for non randomized studies.

	Confounding bias	Selection bias	Classification intervention bias	Deviation from intended intervention	Missing data bias	Measure outcome bias	Selected outcome bias	Overall bias
Mehnert [NCT02054806]	+	+	+	+	+/-	+	+	+
Mehnert [NCT02054806]	+	+	+	+	+/-	+/-	+/-	+
Strosberg [NCT02628067]	+	+	+	+	+/-	+	+	+
Yao [NCT02955069]	+	+	+	+	+/-	+	+	+/-
Yao [NCT02955069]	+	+	+	+	+/-	+	+	+/-
Patel [NCT02834013]	+	+	+	+	+/-	+	+/-	+
Lu [NCT03167853]	+/-	+	+	+	+	+	+	+
Vijayvergia [NCT02939651]	+/-	+	+	+	+	+	+/-	+
Halperin [NCT03074513]	+/-	+	+	+	-	-	+/-	+
Halperin [NCT03074513]	+/-	+	+	+	-	-	+/-	+
Zhang [NCT03167853]	+/-	+	+	+	+/-	+	+/-	+
Fottner [NCT03352934]	+/-	+	+	+	+	+	+/-	+

Mulvey [NCT03136055]	+	+	+	+	+	+	+/-	+
Frumovitz [NCT02721732]	+	+	+	+	+	+	+	+
Rodriguez-Freixinos [NCT03278405]	+	+	+	+	+	+	+	+
Klein [NCT02923934]	+	+	+	+	+	+	+	+
Capdevila [NCT03095274]	+	+	+	+	+	+	+/-	+

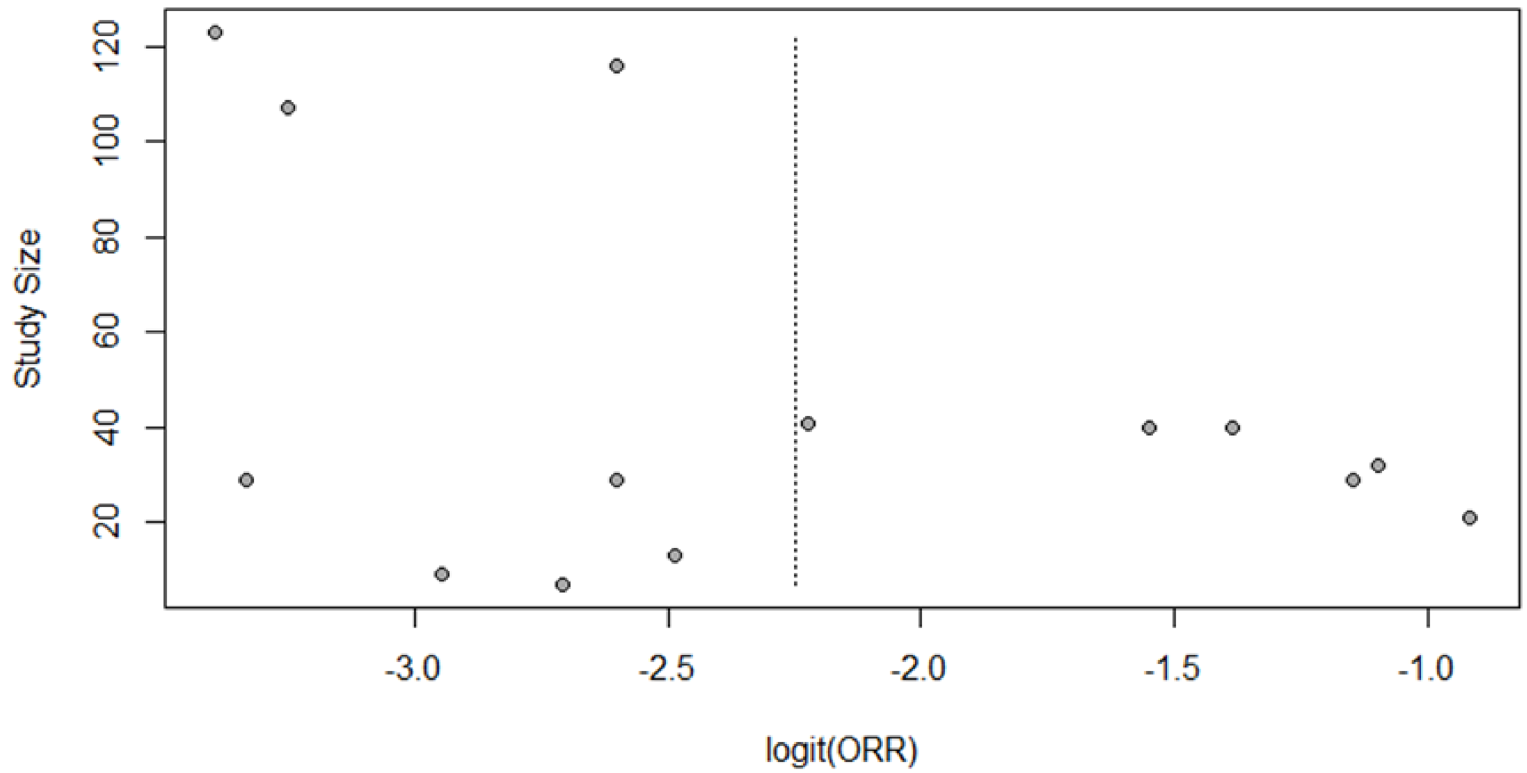

Figure S1: Funnel plot of overall response rate (ORR) for publication bias.
